# Sleep and eye disease: A review

**DOI:** 10.1111/ceo.14071

**Published:** 2022-03-16

**Authors:** Samantha S. Y. Lee, Vinay K. Nilagiri, David A. Mackey

**Affiliations:** ^1^ Centre for Ophthalmology and Visual Science (incorporating the Lions Eye Institute) University of Western Australia Perth Western Australia Australia; ^2^ Centre for Eye Research Australia, University of Melbourne Royal Victorian Eye and Ear Hospital East Melbourne Victoria Australia; ^3^ School of Medicine, Menzies Research Institute Tasmania University of Tasmania Hobart Tasmania Australia

**Keywords:** age‐related macular degeneration, eye disease, glaucoma, sleep, sleep apnoea

## Abstract

There is a growing body of literature on the effects of sleep disorders, in particular obstructive sleep apnoea (OSA), on ocular health, with consistent evidence of an increased risk of floppy eyelid syndrome, non‐arteritic anterior ischaemic optic neuropathy, diabetic macular oedema, and other retinal vasculature changes in individuals with OSA. However, reports on OSA's associations with glaucoma, papilloedema, diabetic retinopathy, central serous chorioretinopathy, and keratoconus have been conflicting, while links between OSA and age‐related macular degeneration have only been described fairly recently. Despite numerous suggestions that OSA treatment may reduce risk of these eye diseases, well‐designed studies to support these claims are lacking. In particular, the ocular hypertensive effects of continuous positive airway pressure (CPAP) therapy for OSA requires further investigation into its potential impact on glaucoma risk and management. Reports of ocular surface complications secondary to leaking CPAP masks highlights the importance of ensuring good mask fit. Poor sleep habits have also been linked with increased myopia risk; however, the evidence on this association remains weak.

## 1 INTRODUCTION

Despite spending one‐third of our lives sleeping, the reason we need sleep is yet unclear. Nonetheless, there is an abundance of evidence that a myriad of bodily functions occur during sleep, including clearance of neurotoxins accumulated during wakefulness, and suppression of catabolism, among other processes,^1^, ^2^ which may maintain homeostasis.

During sleep, the closed eyelid provides a mechanical barrier between the ocular surface and the external environment, while rapid eye movement (REM) promotes transfer of aqueous humour, which has been suggested to increase nutrient supply to the cornea,^3^ compensating for reduced tear production during prolonged eye closure.^4^ Thus, sleep disorders may impact on ocular health, and this was summarised by McNab^4^, ^5^ more than a decade ago. Since then, several large studies and novel findings on this issue have been reported. The current article builds on McNab's previous reviews,^4^, ^5^ discusses the latest findings and controversies on the associations between sleep and eye health, and highlights areas of future research. In particular, we focus on the association between obstructive sleep apnoea (OSA) and glaucoma, which has been the subject of debate in recent years.

## 2 OBTRUCTIVE SLEEP APNOEA

OSA is characterised by episodes of cessation in breathing (called apnoea) or overly shallow breathing (hypopnoea) during sleep due to a mechanical resistance in the upper airway. OSA is the most common sleep disordered‐breathing condition and significantly increases the risk of several cardiovascular disorders, including hypertension and transient ischaemic attack. Moreover, poor‐quality sleep caused by OSA increases fatigue, cognitive impairments, and risk of motor vehicle crash.^4^ OSA has also been linked to increased risk of several eye diseases^4^, ^5^ and treatment for OSA have been suggested to reduce this risk, although there have been limited explorations to test this hypothesis. With the rising prevalence of OSA globally, it is important to understand how this may impact on patients' ocular health and their risk of eye disease.

## 3 PRIMARY OPEN‐ANGLE GLAUCOMA

A possible link between OSA and glaucoma has seen growing interest in the last decade, with four meta‐analyses^6^, ^7^, ^8^, ^9^ published in the mid‐2010s on the associations between the two conditions, all of which concluded that sleep apnoea is associated with glaucoma. The pooled odds ratios (ORs) for glaucoma in patients with sleep apnoea ranged from 1.4 to 2.5. However, as pointed out by two^7^, ^9^ of the four published meta‐analyses, the results were based on statistical outcomes that were unadjusted for potential confounders and studies were generally not of high quality. More recent and large well‐designed cohort or population‐based studies have since failed to find significant associations between OSA and glaucoma, especially after accounting for co‐morbidities.^10^, ^11^, ^12^, ^13^

Instead of relying on the presence of glaucoma, some studies explored associations of sleep apnoea with measures of glaucoma‐related endophenotypes, such as the peripapillary retinal nerve fibre layer (pRNFL) thickness, intraocular pressure (IOP), and visual field defects. Findings from most of these studies supported a link between sleep apnoea and thinner pRNFL, higher IOP or poorer visual fields.^14^, ^15^, ^16^, ^17^, ^18^, ^19^ Five meta‐analyses^20^, ^21^, ^22^, ^23^, ^24^ further noted thinner global pRNFL by 2 to 4 μm in patients with sleep apnoea compared with controls. However, these pRNFL meta‐analyses suffer from similar limitations as the glaucoma ones above—most of the analysed studies did not adjust for potential confounders. To address the issue of confounding variables, we recently examined over 800 young adults free of systemic and ocular disease, and found that those with OSA had thinner pRNFL at the superotemporal and inferotemporal sectors relative to controls.^18^ Importantly, the analysis was adjusted for axial length, which may influence pRNFL thickness measurement, and is unaffected by age given the narrow age range of the study sample (18–22 years). However, the effect size was small, with only a 4‐μm difference in pRNFL thickness between groups.

As part of this review, we performed our own meta‐analysis on the link between OSA and glaucoma or pRNFL thickness. To overcome the potential limitation of pooling results from unadjusted studies, separate meta‐analyses were conducted on studies that did not adjust for co‐morbidities (particularly hypertension and diabetes) or other potential confounders (e.g., age), and those that have adjusted for potential confounders. The detailed methodology and summary statistics are in Note 1 in Data S1. Based on 11 unadjusted studies, the pooled odds for glaucoma is significantly increased in individuals with sleep apnoea [OR = 1.16; 95% confidence interval (CI) = 1.05–1.28]. However, this was not significant based on the results of three studies that adjusted for potential confounders. The pooled hazard ratio (HR) for glaucoma was significantly elevated and the pRNFL thickness was decreased in patients with sleep apnoea compared with controls (HR = 1.2, standardised mean difference = 2.2 μm), and these outcomes were similar between the adjusted and unadjusted studies. The detailed statistical outcomes are presented in Note 2 in Data S1. However, as we describe in Note 3 in Data S1, many of these studies, particularly the pRNFL ones, are still of generally low quality, mostly due to inappropriate selection of control participants.

It has been suggested that the hypoxia and hypoperfusion induced by OSA may increase the risk of optic neuropathy. Therefore, normal‐tension glaucoma (NTG) could be a more likely manifestation of OSA than the high‐tension variant.^4^, ^5^, ^19^ This may explain why significant associations between OSA and glaucoma have been more commonly noted in Asian studies,^19^, ^25^, ^26^ where NTG predominates over primary open‐angle glaucoma (POAG) cases,^27^ than in Western populations.^12^, ^28^, ^29^ However, in a data‐linkage study, Stein et al.^12^ reported that the link between OSA and NTG is even weaker than with POAG, although the authors acknowledged that the relatively small numbers of patients within their sample could have limited the statistical power to find a strong association.

Given the presumptive ischaemia of the optic disc induced by OSA, we may also expect glaucoma progression to be faster in patients with OSA than those without respiratory disease. Indeed, studies have reported that patients with OSA tend to have faster POAG progression than POAG cases without OSA.^30^, ^31^ In a retrospective study comprising 32 patients with POAG, Fan et al.^30^ reported that those with moderate or severe OSA (*n* = 17) had an 8‐fold increase in risk of pRNFL thinning than those with no or mild OSA (*n* = 15), after adjusting for age, sex, body‐mass index (BMI), and co‐morbidities. Over a 3‐year period, Wozniak et al.^31^ similarly reported that the rate of global pRNFL loss in POAG patients with OSA was almost double the rate in those without OSA (−1.1 vs. −0.6 μm/year), after adjustments for potential confounders.

### 3.1 Possible effects of OSA treatment

One of the earliest evidence of the potential for OSA treatment to slow glaucoma progression comes from Kremmer et al.,^32^, ^33^ who reported cases of continued NTG progression in spite of optimal IOP control, and disease progression only slowed down after commencement of continuous positive airway pressure (CPAP) therapy for newly diagnosed sleep apnoea. CPAP therapy, the mainstay of OSA treatment, is highly effective in alleviating upper airway collapse.^34^ Thus, in theory, it should also improve optic nerve perfusion and reduce POAG risk. Findings from a handful of small studies have supported this notion.^35^, ^36^, ^37^ Himori et al.^35^ reported that patients with OSA and POAG had slower rates of visual field loss after undergoing an initial CPAP therapy. However, this study was limited by its small sample size (*n* = 17) and lack of a control group. Moreover, it was unclear how many nights or sessions of CPAP therapy were performed before the visual field effects were seen and how long the effects lasted. Other studies^36^, ^37^ also reported increases in pRNFL thickness, macular thickness or visual field sensitivity after 3–6 months of CPAP therapy. However, these studies also lacked control groups and the improved measures, especially in visual fields, could simply be due to a learning effect.

While CPAP therapy potentially improves optic disc perfusion, its use is known to elevate IOP,^38^, ^39^, ^40^ which may paradoxically increase the risk of POAG or worsen existing disease. As a result, some authors have suggested that patients with glaucoma or those at high risk of glaucoma using CPAP therapy should be closely monitored.^38^, ^39^, ^40^ In a retrospective study involving over 12 000 patients, Chen et al.^26^ reported that CPAP‐treated and untreated patients with OSA had similar levels of increased glaucoma risk, relative to a comparison cohort (HR = 1.65 and 2.15, respectively). However, those who had undergone surgical treatment did not have an elevated risk of glaucoma compared with the controls, suggesting that surgical treatment for OSA may be more beneficial than CPAP therapy in regards to glaucoma risk. In 108 patients with OSA, Lin et al.^37^ noted improvements in visual fields measures and thickening of the macula 6 months after surgical treatment for OSA. However, like the CPAP studies above, this study lacked a control group.

### 3.2 POAG and OSA: summary

From the existing literature and the meta‐analysis reported in this article, there appears to be an association between OSA and increased glaucoma risk. However, even if this link is statistically significant, its clinical significance is unclear. Currently, a more relevant research question may be whether there is any value, or detrimental effects, of OSA treatment of glaucoma progression or risk. Studies that have explored this to date have mostly been of inadequate quality, with small sample sizes and failing to involve control groups. Admittedly, this is a challenging research question to address fully because it is unethical to test in a randomised controlled trial (given the known systemic benefits of OSA treatment). Nonetheless, strong epidemiological studies comprising appropriate control groups and sample sizes may suffice.

## 4 OTHER OPTIC NEUROPATHIES

### 4.1 Non‐arteritic anterior ischaemic optic neuropathy

The presumed optic nerve vascular dysregulation, including hypercapnia, induced by OSA has also been suggested to increase the risk of non‐arteritic ischaemic optic neuropathy (NAION), with a meta‐analysis^41^ estimating that the odds of NAION is increased 6‐fold in those with OSA, compared with controls. However, this meta‐analysis, along with its included studies,^42^, ^43^, ^44^, ^45^, ^46^ may be limited by small sample sizes due to the low prevalence of NAION, which affects only about 2–10 per 100 000 people over 50 years old.^47^, ^48^ Nonetheless, a significant link between OSA and NAION has been consistently reported,^9^, ^42^, ^43^, ^44^, ^45^, ^46^, ^49^, ^50^ suggesting a true association between the two conditions. This was supported by two recent large studies, which reported HRs of 1.7–3.8 for NAION in patients with OSA.^49^, ^50^ Interestingly, the retrospective cohort of over 42 000 participants by Sun et al.^49^ reported that younger patients with OSA (30–39 years) had significantly elevated risk of incident NAION compared with controls (HR = 6.3), but this association was not seen in older participants. The reason for this observation is unclear, although the authors^49^ suggested the possibility that younger patients may have more severe OSA and thus had more severe ocular dysregulation that their older counterparts, or that other risk factors for NAION may come into play with older age and thus the association becomes insignificant in older age groups.

Evidence that treatment for OSA using CPAP therapy may reduce the risk of incident NAION is promising. In a retrospective review of over 2 million clinical records, Stein et al.^12^ reported that untreated patients with sleep apnoea (not specific to OSA) had 16% increased risk of developing NAION relative to those without sleep apnoea, after adjusting for potential confounders including age and co‐morbidities. Those treated with CPAP therapy, on the other hand, did not have elevated NAION risk relative to controls. In another small study of 67 patients with unilateral NAION and OSA, Aptel et at.^51^ reported that those with poor compliance to CPAP therapy had significantly higher risk of a second eye involvement, with a HR of 5.5. Given the importance of preserving the fellow eye in patients with NAION, it may be prudent to consider addressing any undiagnosed or untreated OSA in all patients with NAION. Nevertheless, given the limited findings on the value of OSA treatment in patients with or at risk of NAION, further studies should be undertaken to ascertain these treatment benefits.

### 4.2 Papilloedema

Several hypotheses link papilloedema or idiopathic intracranial hypertension (IIH) to OSA. These include increased central arterial and venous pressure, cerebral vasodilation and increased cerebral blood flow due to hypercapnia and hypoxia, and obesity‐induced raised intra‐abdominal pressure leading to reduced venous return from the brain, all or some of which may result from OSA and could raise intracranial pressure (ICP),^52^ leading to papilloedema. Indeed, in patients with OSA, frequent episodes of ICP elevation during sleep that coincide with apnoeic events, along with decreased oxygen saturation levels, have been noted.^53^ However, reports of a significant link between these conditions have been conflicting. In a cohort of over 2 million people, Stein et al.^12^ reported HRs of 2.0 and 1.3 for papilloedema and IIH, respectively, in patients with untreated OSA relative to controls. Interestingly, CPAP‐treated patients with OSA did not have significantly increased risks of incident IIH, but still had elevated risk of papilloedema compared with controls (HR = 2.1). A small case–control study further reported that men with IIH were more likely to have OSA symptoms or a previous OSA diagnosis (OR = 4.4) than those without ocular problems. On the other hand, studies in which polysomnographies were conducted on patients with newly diagnosed intracranial hypertension did not find that the prevalence of OSA was any higher than that in the general population.^54^, ^55^, ^56^ Peter et al.^57^ similarly failed to find any cases of papilloedema in 95 patients with recently diagnosed OSA.

Rather than having a direct causal link, it could be argued that the relationship between OSA and IIH or papilloedema is related to shared risk factors. In particular, obesity is a well‐established risk factor for both OSA and IIH, with management of both conditions involving weight loss.^58^, ^59^ However, even after adjusting for obesity, Stein et al.^12^ still found significantly higher incidence rates of IIH and papilloedema in patients with OSA. In a weight loss intervention trial, Yiangou et al.^55^ noted a significant association between improvements in OSA status, as quantified using apnoea‐hypopnoea index, and reduced ICP or papilloedema over 12 months, even after accounting for BMI. These suggest that the association between OSA and IIH or papilloedema, if any, may not be fully attributed to obesity as a common risk factor. Given the conflicting reports on the associations between these conditions, and the limited studies on the value of OSA treatment on papilloedema and IIH, further explorations are warranted to better understand this link.

## 5 CHORIORETINAL DISEASE

### 5.1 Central serous chorioretinopathy

Central serous chorioretinopathy (CSC) and OSA have been suggested to share certain underlying pathophysiological mechanisms, for example, increased sympathetic activity and elevated serum cortisol concentrations via activation of the hypothalamic–pituitary–adrenal axis.^60^ Moreover, both conditions share risk factors such as male sex and hypertension.^61^ However, reports on a direct link between these conditions have been inconsistent. Recent population‐based studies^62^, ^63^ reported that participants with sleep apnoea had a slightly higher incidence of CSC by 11%–20% compared to controls. Additionally, CPAP‐treated patients with OSA had about half the risk of CSC than untreated patients.^62^ A meta‐analysis^64^ of six studies also yield an OR for OSA of 1.6 in patients with CSC, relative to controls. However, half of the studies^65^, ^66^, ^67^ included in that meta‐analysis only defined OSA cases as those having a ‘high risk’ of OSA. The 10‐question survey used in these studies (known as the Berlin Questionnaire) includes 5 questions on tiredness after waking (indicating poor sleep quality) and high blood pressure, and an individual would be classified as ‘high‐risk’ for OSA with a positive response to some of these questions. However, poor sleep quality could be an indicator of stress, which, together with high blood pressure, predisposes to CSC. Thus, using such questionnaires to screen for OSA is a fundamental methodological flaw in patients with CSC, as half of the questions are screening for risk factors of CSC. Some data‐linkage population‐based studies that do not rely on questionnaires have found no significant association between OSA and CSC.^68^, ^69^

Nonetheless, these do not rule out a possibility of an indirect link between the conditions. Stress, a major risk factor for CSC, could be exacerbated by poor quality sleep, which may be worsened by OSA. Thus, improving sleep quality in patients with OSA, for example, by using CPAP therapy, may be beneficial for those at high risk of CSC. This has been supported by reports of an association between CPAP therapy use and reduced risk or improvements in CSC.^62^, ^70^

### 5.2 Diabetic retinopathy

The rise in inflammation and oxidative stress in OSA has been suggested to affect energy metabolism, increase insulin resistance and dysglycaemia, and thus increase the risk of type II diabetes.^71^, ^72^, ^73^ Even among non‐diabetic individuals, Steiropoulos et al.^72^ noted that lower oxygen saturation during sleep was associated with higher insulin levels. While some of the link between the two conditions may be mediated by common risk factors, most notably obesity, even lean or non‐obese individuals with OSA exhibit higher levels of insulin than age‐, sex‐, and BMI‐matched controls,^74^, ^75^ suggesting that OSA could be an independent risk factor for diabetes.

While there has been consistent evidence supporting a link between OSA and diabetes, findings on an independent association between OSA and diabetic retinopathy (DR) have been inconsistent, with even meta‐analyses arriving at different conclusions.^76^, ^77^ Leong et al.^77^ highlighted that many previous studies that have reported a significant association between OSA and prevalence of DR did not adjust for potential confounders. However, adjusted studies have found that the presence of OSA is associated with more severe DR^78^ or progression in DR.^79^, ^80^ Cross‐sectional studies^81^, ^82^ have reported that CPAP therapy use may reduce DR rates or progression of DR, although this has yet to be verified or disputed by prospective longitudinal studies. Given that CPAP therapy use for OSA treatment may be beneficial for controlling systemic glycemic and insulin levels,^82^ it is plausible that it could also improve ocular outcomes in patients with diabetes. This effect may be more apparent in patients with macular disease, including diabetic macular oedema (DMO), as discussed in the following sub‐section.

### 5.3 Age‐related macular degeneration and macular oedema

In the mid‐2010s, Schaal et al.^83^, ^84^ noted that patients with age‐related macular degeneration (AMD) or DMO were more likely to have poor response to anti‐vascular endothelial growth factor (VEGF) therapy if they had symptoms of OSA or untreated OSA. In 38 patients with OSA and anti‐VEGF injections for exudative AMD, the patients with untreated OSA required double the number of injections compared with those treated with CPAP (mean of 16 vs. 8 injections) to reduce the macular oedema. Moreover, the untreated OSA group had poorer final visual acuity and thinner maculas than the CPAP‐treated group after completing the anti‐VEGF injection regimens (visual acuity: 0.7 vs. 0.3 logMAR; macular thickness: 322 vs. 254 μm), despite having similar baseline measures prior to anti‐VEGF therapy.^84^ In another case–control study, the authors^83^ noted that high proportion of patients with AMD with poor response to anti‐VEGF therapy (79%) had symptoms and other risk factors of OSA (e.g., obesity, high blood pressure), compared with less than half of participants in the other groups (including participants with no AMD, non‐exudative AMD, or with exudative AMD but had good response to anti‐VEGF treatment). Similarly, a larger proportion of patients with DMO who required two or more consecutive anti‐VEGF injections had symptoms and risk factors of OSA than those who required only one injection (78%–88% vs. 50%).

The mechanism underlying the poorer treatment response is unclear, although there was a suggestion in an editorial article that an upregulation of VEGF levels due to the hypoxia induced by OSA may offset the effects of the anti‐VEGF therapy.^85^ This, by extension, could mean that the presence of OSA does not merely reduce anti‐VEGF treatment response, but may also increase the risk of exudative AMD or DMO. Indeed, a link between OSA and DMO have been reported.^86^, ^87^ In patients with Type II diabetes, Chiang et al.^86^ found a higher incidence rate of DMO in those with OSA than those without OSA (HR = 3.0), while another study reported that those with DMO were more likely to have higher apnoea‐hypopnoea index and lower oxygen saturation levels as measured using polysomnographies.^87^ In the United Kingdom, Keenan et al.^29^ reported on a large data‐linkage study that found an elevated risk of AMD by 44% in patients with OSA, relative to controls. Recently, we further reported that the hazard of AMD is raised by 33%–39% in two large‐scale cohorts, after accounting for potential confounders including co‐morbidities.^88^ Given the relative novelty of the reported associations between OSA and AMD, and importance of AMD as the leading cause of blindness and visual impairment, further studies on the link between these conditions are warranted. The benefits of OSA treatment on AMD risk and visual outcome, is particularly worthy of exploration.

### 5.4 Other retinal vascular changes

Given the association between OSA and vascular diseases such as systemic hypertension, similar vascular manifestations of OSA in the eye could be expected. In a cross‐sectional study of 115 consecutive individuals who underwent an overnight polysomnography,^89^ participants with moderate or severe OSA had higher rates of arteriosclerotic changes and increased vessel tortuosity compared with controls, even after adjusting for the presence of systemic hypertension. The study also reported other changes reminiscent of hypertensive retinopathy, including significantly smaller arteriovenous ratio, increased retinal arteriolar narrowing, reduced arteriolar and venous pulsation, as well as reduced vessel calibre, with increasing OSA severity after correcting for systemic blood pressure. It is unclear why retinal vasculature changes may occur independently of systemic blood pressure measures. Tong et al.^89^ suggested an increase in intracranial pressure and venous pressure in OSA can mechanically hamper retinal blood flow, resulting in some of the observed retinal vasculature changes. Time of day at which the blood pressure measurement and retinal vessel imaging were performed, as well as use of anti‐hypertensive medications, may also affect the observed relationship between OSA and retinal vasculature measures.

An increase in risk of systemic hypertension in individuals with OSA is hypothesised to be due to the OSA‐induced hypoxia and hypercapnia, which result in sympathetic activation, oxidative stress, vessel endothelial damage, and increased blood coagulation, all of which are drivers of vascular disease.^90^, ^91^ In particular, the state of hypercoagulability increases the risk of retinal vein occlusion (RVO). Indeed, a longitudinal population‐based study in Taiwan reported that individuals with sleep apnoea (not specific to OSA) had almost double the rates of incident RVO compared with controls, after correcting for age, sex, and co‐morbidities.^92^ It is unclear how OSA treatment, using CPAP therapy or otherwise, may impact the risk of retinal vascular changes or risk of RVO. Further studies are warranted to explore this.

## 6 ANTERIOR SEGMENT

### 6.1 Floppy eyelid syndrome and corneal changes

Floppy eyelid syndrome (FES) is perhaps the most well‐described ocular disorder to have an association with OSA. People with FES have extremely lax eyelids, leading to an increased ease of eyelid eversion and episodes of spontaneous eversion of the eyelid which tends to happen during sleep. The eversion of the eyelids increases ocular surface exposure to desiccation and external pollutants, which may be especially problematic during sleep as the palpebral conjunctiva and cornea can rub against the pillow.^4^ As a result, people with FES almost always present with superficial punctate keratopathy,^93^ with associated cases of corneal scarring, neovascularisation, thinning, recurrent erosions, and even perforation reported.^93^, ^94^, ^95^, ^96^, ^97^

While treating OSA has been suggested to reduce the risk of or even reverse FES,^4^, ^5^ no randomised or non‐randomised controlled trials have been published to support this. Viera et al.^98^ observed 34 patients with newly‐diagnosed OSA and FES and found that FES was reversed in about half of the patients after 6 months of CPAP therapy. However, the lack of a control group in the study renders its evidence inconclusive. Moreover, in a study of 89 patients with OSA, Kadyan et al.^28^ failed to find any significant difference in upper or lower lid laxity between CPAP‐treated and untreated patients.

### 6.2 Keratoconus

Corneal changes occurring with FES, such as reduced corneal hysteresis and increased tendency to eye rubbing, have been suggested to predispose to keratoconus.^99^, ^100^ A case–control study^101^ further reported that patients with sleep apnoea had thinner corneas by 20 μm compared with controls, with increased severity of sleep apnoea associated with thinner corneas. Nonetheless, there have been inconsistent findings on a link between OSA and keratoconus. A meta‐analysis estimated that patients with OSA have 84% increase in odds for keratoconus compared with controls.^102^ A retrospective cohort study^103^ further reported that the presence of OSA slightly increased the odds of keratoconus by 13%. Others have also reported that patients with keratoconus have a high prevalence of OSA (18%–20%) or are at high risk of OSA (12%–53%) as assessed by the Berlin Questionnaire.^104^, ^105^, ^106^ However, based on population‐based estimates, OSA affects up to half of middle‐aged and older adults in the general population.^107^, ^108^ Thus, an OSA prevalence of 18%–20% among patients with keratoconus may not be significantly higher than in a general population. Several other larger studies have also failed to find that OSA is a significant risk factor for keratoconus.^109^, ^110^, ^111^, ^112^ Like OSA's association with POAG, any link with keratoconus is likely to be weak at best, given the inconsistent reports and small increase in odds for keratoconus in patients with OSA.

### 6.3 Ocular surface complications related to CPAP therapy use

CPAP masks have to be fitted to the face such that there is minimal air leak from the mask during its use. Because of the proximity of the CPAP masks to the eye, leaking CPAP masks could result in prolonged airflow to the eye during sleep. Cases of unilateral conjunctivitis,^113^, ^114^ reactivation of recurrent corneal erosion,^115^ and lipid keratopathy^116^ believed to have been due to leaky CPAP have been reported. In these cases, improvements in clinical signs and symptoms followed the cessation of CPAP use or commencement of ocular patching during sleep. There was even one report of a trabeculectomy bleb‐related endophthalmitis, which the authors believed to have been caused by a spread of bacteria from the respiratory tract to the eye via the CPAP mask.^117^ A more systematic study found that about one‐fifth of CPAP therapy users had previous episodes of conjunctivitis secondary to CPAP mask leaks.^28^ However, on average, there were no significant difference in rates of ocular irritation symptoms between CPAP and non‐CPAP users, and CPAP users even had longer tear break‐up time than non‐users. This suggests that, when fitted well, CPAP therapy may be beneficial for the ocular surface. However, the case reports are a reminder that ill‐fitting masks have the potential to inflict clinically significant and even potentially sight‐threatening damage, although these are uncommon. It may be prudent for clinicians to ask their patients who are on CPAP‐therapy about mask fitting, leaking masks, and symptoms of ocular irritation, to ensure that suggestions to improve mask fitting are made before any more severe ocular complications can occur.

## 7 OTHER POTENTIAL LINKS BETWEEN SLEEP AND EYE DISORDERS

### 7.1 Myopia

With the world undergoing a myopia epidemic, there is increased interest in finding effective methods of myopia control and identifying novel risk factors for myopia. It has been suggested that our circadian rhythm mediates the diurnal variations of the axial length, which is longest around mid‐day and shortest around midnight, and choroidal thickness, which is thinnest in the morning and thickest at night.^118^, ^119^ Given that our circadian rhythm is regulated by light exposure, it is conceivable that disruptions to sleep duration or quality may adversely affect the normal diurnal variations in axial length and choroidal thickness, resulting in refractive error.^118^

Reports on the relationship between sleep duration or quality and myopia development have been conflicting,^120^, ^121^, ^122^, ^123^, ^124^, ^125^, ^126^, ^127^ although this may be attributed to the cross‐sectional design or retrospective data collection of many of these studies, which reduces the power of these studies to find any true causative relationship. Recently, we explored if a link between sleep quality during childhood was associated with future myopia development in a prospective cohort study of over 1000 adults, but also failed to find a significant association.^127^ Other longitudinal prospective studies^123^, ^125^, ^128^ similarly failed to find such associations, suggesting that the link between myopia and sleep duration or quality, if any, is not clinically significant.

## 8 CONCLUSIONS AND FUTURE DIRECTIONS

Sleep habits and disorders may have significant influence on eye health and risk of eye disease. As the prevalence of OSA is likely to increase over the decades with increasing rates of obesity, we could expect more people to be at risk of the eye diseases discussed in this article. While there is evidence that treatment of OSA can improve NAION or reduce the risk of a second eye involvement, there has been a paucity of studies on how OSA treatment may reduce the risk or severity of many of the other associated eye diseases. In particular, more attention should be directed towards alleviating the OSA‐associated increase in risks of POAG and AMD, as these are the two leading causes of irreversible visual impairment worldwide.^129^ Further research into the potential benefits, or lack thereof, of CPAP or surgical treatment for OSA on these conditions are warranted.

## CONFLICT OF INTEREST

The authors declare no conflicts of interest.

## Supporting information


**Appendix** **S1**: Supporting Information
