# Supplementary material for: Sleep and eye disease: A review
Source: Clin Exp Ophthalmol. 2022 Mar 16;50(3):334–44. doi: 10.1111/ceo.14071 (PMC9544516; doi:10.1111/ceo.14071)
Supplement: Supplementary file 1 — Appendix S1: Supporting Information [file CEO-50-334-s001.docx]

**SUPPLEMENTARY NOTE 1: META-ANALYSIS METHODS**

*Associations between obstructive sleep apnoea (OSA) and glaucoma or peripapillary retinal nerve fibre layer (pRNFL)*

Search strategy: A systemic search of Medline, Web of Science, and Embase was conducted on 28 July 2021. The search terms used were *(((sleep apnoea[MeSH Terms]) OR (sleep apnea[MeSH Terms])) AND ((glaucoma[MeSH Terms]) OR (optic neuropathy[MeSH Terms]) OR (retinal nerve fibre[MeSH Terms]) OR (disc[MeSH Terms]) OR (disk[MeSH Terms]) OR (optic nerve[MeSH Terms]))* on Medline, *((ALL=(sleep apnoea) OR ALL=(sleep apnea)) AND (ALL=(glaucoma) OR ALL=(optic neuropathy) OR ALL=(retinal nerve fibre) OR ALL=(disk) OR ALL=(disc)))* on Web of Science, and *(optic neuropathy/ or glaucoma/ or retinal nerve fibre/ or disc/ or disk) and (sleep apnea/ or sleep apnoea/)* on Embase. The authors additionally searched their own bibliography library and the references lists of studies found.

The title and abstract of each study from the search results were reviewed for pertinence and whether they attempted to answer at least one of the two research questions posed. If considered potentially suitable for inclusion in the current meta-analyses, the full text of the study was obtained and we determined if it met the inclusion criteria.

Study selection: To be included in the meta-analysis, the study had to answer at least one of the two research questions posed and include a control (non-sleep apnoea) group for comparison. For studies that examined the association between glaucoma and sleep apnoea, the glaucoma had to be diagnosed by a physician, self-reported as having been diagnosed by a physician or determined by data-linkage, and the article had to provide the adjusted and/or unadjusted ORs or hazard ratios (HRs) of glaucoma in participants with sleep apnoea. For studies on the association between pRNFL thickness and sleep apnoea, the adjusted and/or unadjusted mean difference in global pRNFL between groups and the 95% confidence interval (CI) or standard error of the mean difference had to be reported. As different studies may have adjusted for different confounding variables, adjusted ORs or HRs had to, at minimum, account for age, sex, as well as the presence of diabetes and systemic hypertension either at the statistical analysis stage or by participant-matching. We considered the inclusion of diabetes and hypertension to be imperative in adjusted OR or HR calculations as these are common systemic conditions and are strongly associated with cardiovascular disease, which has been linked with both sleep apnoea^1^ and glaucoma.^2^

Alternatively, enough data had to be provided by the studies to calculate the ORs or HRs of glaucoma, or the mean difference in pRFNL thickness, and the 95%CI or standard error of these statistical outcomes. Case studies, review articles, editorials, meta-analyses, conference abstracts, non-human studies, and articles that were not published in English were excluded.

Data extraction: The following information were extracted from each paper: (1) last name of the first author, (2) publication year, (2) total sample size, (3) sample size in each subgroup (sleep apnoea/control, glaucoma/no glaucoma), (4) ORs, HRs, or mean difference, (5) the 95%CI, (6) correction for potential confounders (including age, sex, co-morbidities), and (5) age and ethnicity of study participants. If the OR, HR, or mean pRNFL difference was not provided by the study, the uncorrected OR or mean pRNFL difference and its 95%CI was calculated if enough information was provided by the study to do so.

Study quality: Assessment of study quality was based on the Newcastle-Ottawa Scale (NOS),^3^ which is commonly used for observational studies, or a version of the scale adapted for cross-sectional studies.^4^ The NOS evaluates three elements of each study: participant selection, comparability of control and exposure groups, and outcome, with a maximum of 4, 2, and 3 points allocated to each element. The maximum score for case-control and cohort studies is 9, while the maximum possible score for cross-sectional studies is 7. Studies with 7 or more points were considered to be of good quality (meaning that cross-sectional studies had to attain the maximum score to be considered to be of good quality), while 6 points means that a study was of adequate quality. Two authors (SSYL and VKN) graded the studies independently and any discrepancy in grading were then discussed until a consensus is reached.

Statistical analysis: The meta-analyses were conducted on RStudio v3.6.3 (The R Foundation for Statistical Programming, Vienna, Austria; <https://www.r-project.org/>) using the “meta” package, and the level of statistical significance was set at p< 0.05. The most commonly reported statistical outcome in studies on the association between sleep apnoea and glaucoma were ORs and HRs. However, these measures are fundamentally different: ORs are derived from cross-sectional observations and are cumulative over an entire study, while HRs are derived from survival analyses and measures risk over the study period. We thus performed separate analyses to obtain the adjusted and unadjusted pooled OR and HR and their 95%CI. The outcome measure pooled from studies comparing pRNFL thickness between patients with sleep apnoea and controls was the standardised mean difference (SMD) and its 95%CI.

We reported the statistical outcome from fixed or random effects meta-analyses depending on the level of heterogeneity of studies. Heterogeneity between studies was quantified using the *τ^2^* index, which is an estimate of the variance of the distribution of the true effect sizes, and the *I^2^* index, which indicates the proportion of total variation due to heterogeneity.^5,6^ Higher *τ^2^* or *I^2^* value represents higher heterogeneity.

Summary statistics: Of 845 reports from the database search, 46 eligible studies were identified. These, in addition to two further articles from the authors’ bibliography library,^7,8^ were included in the current study. Of these, 15 studies analysed the association between sleep apnoea and glaucoma, and 24 explored the former’s association with pRNFL thickness. One article^9^ reported on two cohort studies separately, and was thus considered as two distinct studies. The study selection process is shown in the flow chart below:


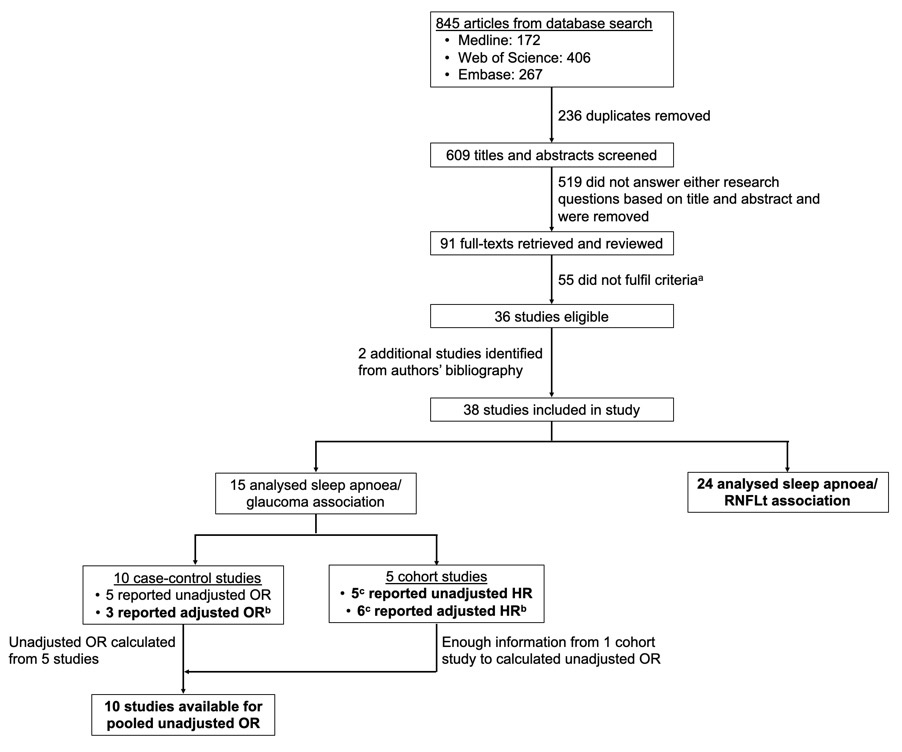


***Selection process for the inclusion of studies in the meta-analysis.*** *HR= hazard ratio; OR= odds ratio; RNFLt= retinal nerve fibre layer thickness; ^a^ 29 studies did not have a control group, 4 studies did not diagnose sleep apnoea from a sleep study, and 22 studies did not provide OR, HR, mean RNFLt difference, or enough information to calculate these measures; ^b^ reported ORs and HRs had to, at minimum, adjusted for age, sex, systemic hypertension, and diabetes; ^c^ one study (Han X et al. 2021) analysed two cohorts separately, thus giving rise to one extra cohort study*

**SUPPLEMENTARY NOTE 2: META-ANALYSES RESULTS**

Sleep apnoea and glaucoma: As seen in the forest plots below, the pooled unadjusted ORs for glaucoma in patients with sleep apnoea was 1.16 (95% CI= 1.05–1.28; p= 0.003), based on the random effects model analysing 11 studies comprising 2,226,224 participants. However, based on the three studies (total n= 7,632 participants) that had adjusted for confounders, both the fixed- and random-effects models revealed an insignificant association between glaucoma and sleep apnoea (pooled OR= 1.26, 95%CI= 0.93–1.71; p= 0.13).


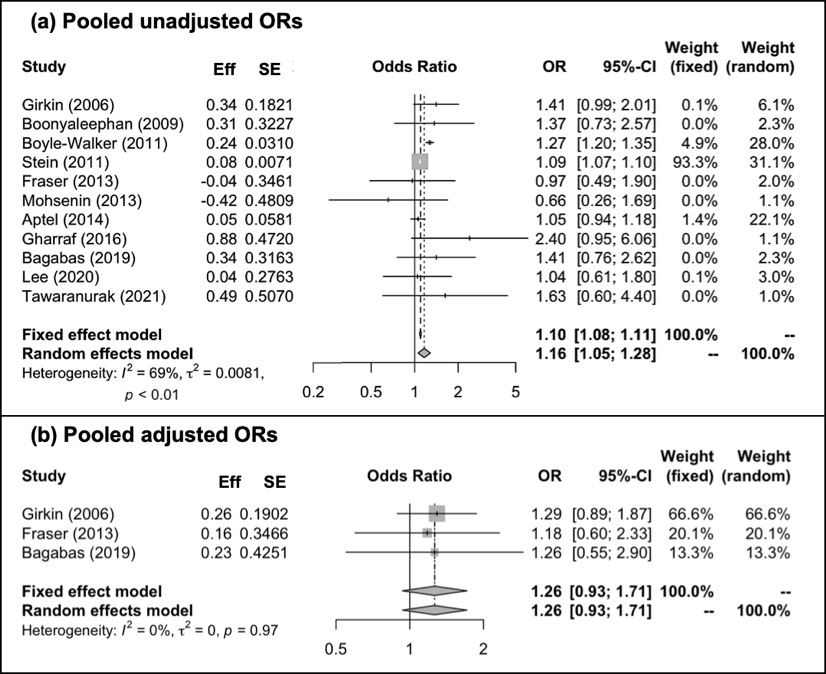


***Forest plot showing the pooled odds ratios of glaucoma with sleep apnoea*** *in studies that have (a) not adjusted and (b) adjusted for co-morbidities. CI= confidence interval; OR= odds ratio; SE= standard error of the effect size.*

Adjusted HRs of glaucoma were reported by six cohort studies with a total of 2,778,471 participants. Five of these studies comprising 2,722,052 participants additionally reported the unadjusted HR. The pooled unadjusted and adjusted HRs were both significantly elevated at approximately 1.20 (95%CI= 1.06–1.35; p= 0.003) based on the random-effects models (see forest plot below).


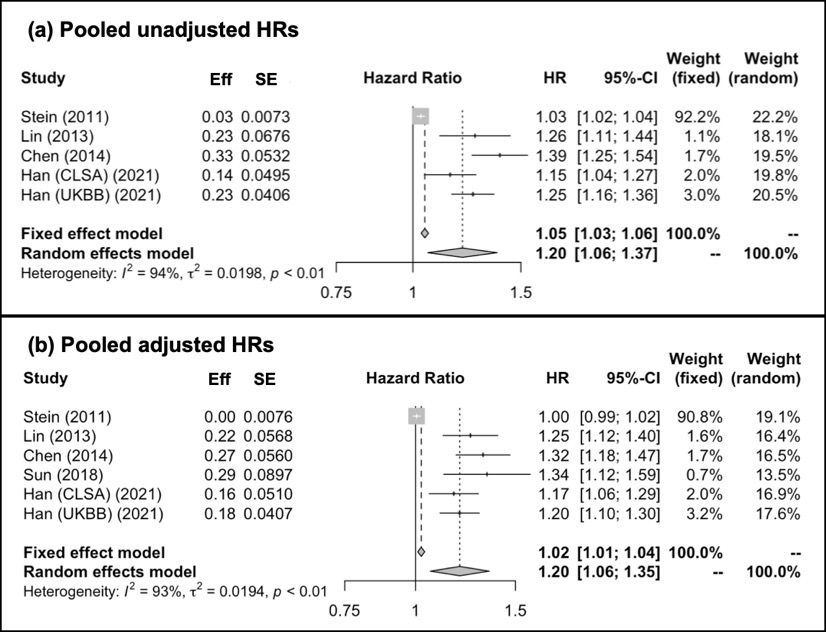


***Forest plot showing the pooled hazard ratios of glaucoma with sleep apnoea*** *in studies that have (a) not adjusted and (b) adjusted for co-morbidities. CI= confidence interval; Eff= effect size; HR= hazard ratio; SE= standard error of the effect size*

Sleep apnoea and retinal nerve fibre layer thickness: The unadjusted difference in global pRNFL thickness between participants with sleep apnoea and controls was extracted from 17 eligible studies comprising 2,332 participants. In addition, seven studies comprising 1,375 participants reported the mean difference in global pRNFL, adjusted for age and co-morbidities. Meta-analyses of both the adjusted and unadjusted studies revealed similar SMD in global pRNFL — participants with sleep apnoea had, on average, thinner global pRNFL by approximate -2.2µm (both adjusted and unadjusted p< 0.001; see forest plot below).


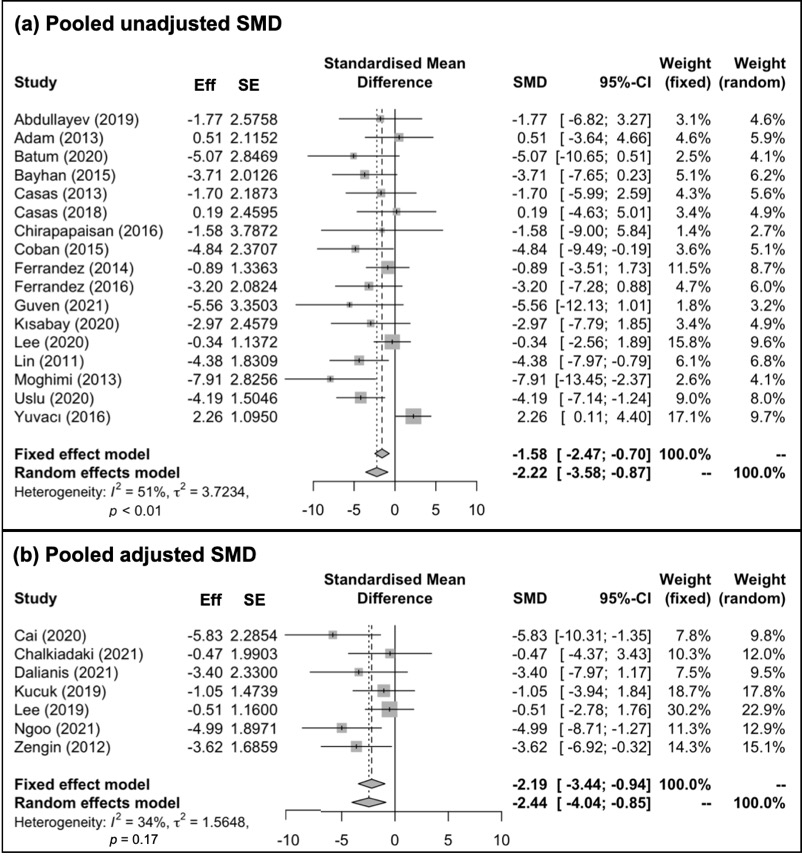


***Forest plot showing the pooled standardised mean difference in global peripapillary retinal nerve fibre layer thickness (µm)*** *in participants with sleep apnoea compared to those without sleep apnoea, (a) without adjustments and (b) with adjustments for co-morbidities. CI= confidence interval; Eff= effect size; SE= standard error of the effect size; SMD= standardised mean difference*

**SUPPLEMENTARY NOTE 3: QUALITY OF STUDIES**

All of the six cohort studies assessing the HRs of glaucoma in patients with sleep apnoea were of good quality, with NOS scores of 8 or 9. Of the 10 case-control studies, only four studies attained a 7 out of 9, which just meets the threshold of good quality.^10-13^ Two studies were of adequate quality (score=6),^14,15^ while the remaining four studies only scored 3–5.^7,8,16,17^ The main domains where studies lost points were (1) selection of controls, where seven studies^7,8,10-12,16,17^ recruited hospital rather than community controls, and (2) non-response rate, where seven case-control studies^7,10,12,14,16,17^ did not report the non-response rate in both the cases and control group. Other common areas of points lost pertained to the “comparability” element of the NOS, with six case-control studies^7,8,13,14,16,17^ failing to control for age and/or the presence of co-morbidities (which also was a criterion for inclusion in the adjusted meta-analysis in the current study).

Studies that assessed the pRNFL thickness were of lower quality than those that reported glaucoma rates, with only one case-control study attaining a NOS score of 7.^18^ Of the 24 studies that assessed pRNFL thickness, three were cross- sectional studies,^14,19,20^ none of which attained the maximum possible NOS score because of the modified NOS for cross-sectional studies. One cross-sectional study^20^ had a score of 6 and thus was considered to be of adequate quality, while the other two failed to meet this threshold. ^14,19^ Seven case-control studies had a score of 6 and were considered to be of adequate quality.^14,21-26^ The main component where points were lost was selection of controls, where most studies recruited controls from hospitals rather than the community or failed to describe the setting in which controls were recruited (n= 20 studies).^21-40^ Eighteen case-control studies did not describe non-response rate of potential participants approached,^22,24,27-40^ while 17 lost points on “representativeness of the cases”, where there was potential selection bias or representativeness was not stated.^19,21,22,24,27-30,32,34-40^ Other domains where points were lost were the “comparability” of cases and controls (not correcting for age and/or co-morbidities) and “method of ascertainment for cases and controls”.

**REFERENCES**

1 Sanchez-de-la-Torre, M., Campos-Rodriguez, F. & Barbe, F. Obstructive sleep apnoea and cardiovascular disease. *Lancet Respir Med* **1**, 61-72, doi:10.1016/S2213-2600(12)70051-6 (2013).

2 Hewitt, A. W., Sanfilippo, P., Ring, M. A., Craig, J. E. & Mackey, D. A. Mortality in primary open-angle glaucoma: 'two cupped discs and a funeral'. *Eye* **24**, 59-63, doi:10.1038/eye.2009.37 (2010).

3 Peterson, J., Welch, V., Losos, M. & Tugwell, P. The Newcastle-Ottawa scale (NOS) for assessing the quality of nonrandomised studies in meta-analyses. *Ottawa: Ottawa Hospital Research Institute*, 1-12 (2011).

4 Patra, J. *et al.* Exposure to second-hand smoke and the risk of tuberculosis in children and adults: a systematic review and meta-analysis of 18 observational studies. *PLoS Med* **12**, e1001835; discussion e1001835, doi:10.1371/journal.pmed.1001835 (2015).

5 Harrer, M., Cuijpers, P., Furukawa, T. A. & Ebert, D. D. *Doing Meta-Analysis With R: A Hands-On Guide*. 1st edn, (Chapman & Hall/CRC Press, 2021).

6 Deeks, J. J., Higgins, J. P., Altman, D. G. & Group, C. S. M. Analysing data and undertaking meta‐analyses. *Cochrane handbook for systematic reviews of interventions*, 241-284 (2019).

7 Boonyaleephan, S. & Neruntarat, C. The association of primary open-angle glaucoma/normal tension glaucoma and obstructive sleep apnea in Thai patients. *วารสาร การ แพทย์ และ วิทยาศาสตร์ สุขภาพ (Journal of Medicine and Health Science)* **15** (2009).

8 Boyle-Walker, M., Semes, L. P., Clay, O. J., Liu, L. & Fuhr, P. Sleep apnea syndrome represents a risk for glaucoma in a veterans' affairs population. *ISRN Ophthalmol* **2011**, 920767, doi:10.5402/2011/920767 (2011).

9 Han, X. *et al.* Associations of sleep apnoea with glaucoma and age-related macular degeneration: an analysis in the United Kingdom Biobank and the Canadian Longitudinal Study on Aging. *BMC Med* **19**, 104, doi:10.1186/s12916-021-01973-y (2021).

10 Aptel, F. *et al.* Association between glaucoma and sleep apnea in a large French multicenter prospective cohort. *Sleep Med* **15**, 576-581, doi:10.1016/j.sleep.2013.11.790 (2014).

11 Bagabas, N. *et al.* Prevalence of Glaucoma in Patients with Obstructive Sleep Apnea. *J Epidemiol Glob Health* **9**, 198-203, doi:10.2991/jegh.k.190816.001 (2019).

12 Fraser, C. L. *et al.* A prospective photographic study of the ocular fundus in obstructive sleep apnea. *J Neuroophthalmol* **33**, 241-246, doi:10.1097/WNO.0b013e318290194f (2013).

13 Girkin, C. A., McGwin, G., Jr., McNeal, S. F. & Owsley, C. Is there an association between pre-existing sleep apnoea and the development of glaucoma? *The British journal of ophthalmology* **90**, 679-681, doi:10.1136/bjo.2005.086082 (2006).

14 Lee, S. S. *et al.* Optic Disc Measures in Obstructive Sleep Apnoea: A Community-based Study of Middle-aged and Older Adults. *Journal of glaucoma* **25**, 337-343(337), doi:10.1097/IJG.0000000000001485 (2020).

15 Tawaranurak, K., Dumkleang, A. & Kiddee, W. Prevalence of Primary Open-Angle Glaucoma in Patients with Obstructive Sleep Apnea Syndrome in Thailand. *JOURNAL OF THE MEDICAL ASSOCIATION OF THAILAND* **104**, 787-793 (2021).

16 Gharraf, H., Zidan, M. H. & ElHoffy, A. Association between obstructive sleep apnea hypopnea syndrome and normal tension glaucoma. *Egyptian Journal of Chest Diseases and Tuberculosis* **65**, 239-249, doi:10.1016/j.ejcdt.2015.11.003 (2016).

17 Mohsenin, A., Mohsenin, V. & Adelman, R. A. Retinal vascular tortuosity in obstructive sleep apnea. *Clinical ophthalmology* **7**, 787-792, doi:10.2147/OPTH.S41795 (2013).

18 Casas, P. *et al.* Visual field defects and retinal nerve fiber imaging in patients with obstructive sleep apnea syndrome and in healthy controls. *BMC Ophthalmol* **18**, 66, doi:10.1186/s12886-018-0728-z (2018).

19 Chirapapaisan, N. *et al.* Diurnal changes in retinal nerve fiber layer thickness with obstructive sleep apnea/hypopnea syndrome. *Int J Ophthalmol* **9**, 979-983, doi:10.18240/ijo.2016.07.07 (2016).

20 Lee, S. S. *et al.* Associations between Optic Disc Measures and Obstructive Sleep Apnea in Young Adults. *Ophthalmology*, doi:10.1016/j.ophtha.2019.04.041 (2019).

21 Chalkiadaki, E. *et al.* Ganglion cell layer thickening in patients suffering from Obstructive Sleep Apnea-Hypopnea syndrome with long Mean Apnea-Hypopnea Duration during sleep. *Int Ophthalmol* **41**, 923-935, doi:10.1007/s10792-020-01648-2 (2021).

22 Dalianis, G. *et al.* Association of obstructive sleep apnea/hypopnea syndrome with glaucomatous optic neuropathy and ocular blood flow. *Exp Ther Med* **21**, 657, doi:10.3892/etm.2021.10089 (2021).

23 Guven, S., Kilic, D. & Bolatturk, O. F. Thinning of the inner and outer retinal layers, including the ganglion cell layer and photoreceptor layers, in obstructive sleep apnea and hypopnea syndrome unrelated to the disease severity. *Int Ophthalmol*, doi:10.1007/s10792-021-01937-4 (2021).

24 Kucuk, B., Sirakaya, E. & Delibas, S. Posterior segment assessment in patients with obstructive sleep apnea syndrome. *Sleep Breath* **23**, 997-1005, doi:10.1007/s11325-019-01837-z (2019).

25 Lin, P. W. *et al.* Decreased retinal nerve fiber layer thickness in patients with obstructive sleep apnea/hypopnea syndrome. *Graefe's archive for clinical and experimental ophthalmology = Albrecht von Graefes Archiv fur klinische und experimentelle Ophthalmologie* **249**, 585-593, doi:10.1007/s00417-010-1544-1 (2011).

26 Zengin, M. O. *et al.* The Relationship Between Obstructive Sleep Apnea Syndrome and Glaucoma. *Turkiye Klinikleri Tip Bilimleri Dergisi* **32**, 990-996, doi:10.5336/medsci.2011-25587 (2012).

27 Abdullayev, A., Tekeli, O., Yanik, O., Acican, T. & Gulbay, B. Investigation of the Presence of Glaucoma in Patients with Obstructive Sleep Apnea Syndrome Using and Not Using Continuous Positive Airway Pressure Treatment. *Turk J Ophthalmol* **49**, 134-141, doi:10.4274/tjo.galenos.2018.88614 (2019).

28 Adam, M. *et al.* The evaluation of retinal nerve fiber layer thickness in patients with obstructive sleep apnea syndrome. *Journal of ophthalmology* **2013**, 292158, doi:10.1155/2013/292158 (2013).

29 Batum, M. *et al.* Evaluation of effects of positive airway pressure treatment on retinal fiber thickness and visual pathways using optic coherence tomography and visual evoked potentials in the patients with severe obstructive sleep apnea syndrome. *International Ophthalmology* **40**, 2475-2485, doi:10.1007/s10792-020-01426-0 (2020).

30 Bayhan, H. A., Aslan Bayhan, S., Intepe, Y. S., Muhafiz, E. & Gurdal, C. Evaluation of the macular choroidal thickness using spectral optical coherence tomography in patients with obstructive sleep apnoea syndrome. *Clin Exp Ophthalmol* **43**, 139-144, doi:10.1111/ceo.12384 (2015).

31 Casas, P. *et al.* Retinal and optic nerve evaluation by optical coherence tomography in adults with obstructive sleep apnea-hypopnea syndrome (OSAHS). *Graefe's archive for clinical and experimental ophthalmology = Albrecht von Graefes Archiv fur klinische und experimentelle Ophthalmologie* **251**, 1625-1634, doi:10.1007/s00417-013-2268-9 (2013).

32 Coban, D. T. *et al.* GLAUCOMA AND OTHER OCULAR FINDINGS IN OBSTRUCTIVE SLEEP APNEA SYNDROME. *Acta Medica Mediterranea* **31**, 363-369 (2015).

33 Cai, Y. *et al.* Quantitative evaluation of retinal microvascular circulation in patients with obstructive sleep apnea-hypopnea using optical coherence tomography angiography. *Int Ophthalmol* **40**, 3309-3321, doi:10.1007/s10792-020-01518-x (2020).

34 Ferrandez, B. *et al.* Retinal sensitivity is reduced in patients with obstructive sleep apnea. *Invest Ophthalmol Vis Sci* **55**, 7119-7125, doi:10.1167/iovs.14-14389 (2014).

35 Ferrandez, B. *et al.* Assessment of the retinal nerve fiber layer in individuals with obstructive sleep apnea. *BMC Ophthalmol* **16**, 40, doi:10.1186/s12886-016-0216-2 (2016).

36 Kisabay Ak, A. *et al.* Evaluation of retinal fiber thickness and visual pathways with optic coherence tomography and pattern visual evoked potential in different clinical stages of obstructive sleep apnea syndrome. *Doc Ophthalmol* **141**, 33-43, doi:10.1007/s10633-020-09749-0 (2020).

37 Moghimi, S. *et al.* Retinal nerve fiber thickness is reduced in sleep apnea syndrome. *Sleep Med* **14**, 53-57, doi:10.1016/j.sleep.2012.07.004 (2013).

38 Ngoo, Q. Z., A, N. F., A, B. & Wh, W. H. Evaluation of Retinal Nerve Fiber Layer Thickness and Optic Nerve Head Parameters in Obstructive Sleep Apnoea Patients. *Korean J Ophthalmol* **35**, 223-230, doi:10.3341/kjo.2020.0019 (2021).

39 Uslu, H., Kanra, A. Y. & Sarac, S. Structural assessment of the optic nerve in patients with obstructive sleep apnea syndrome: Case-control study. *Eur J Ophthalmol*, 1120672120926859, doi:10.1177/1120672120926859 (2020).

40 Yuvaci, I. *et al.* Evaluation of posterior ocular changes using enhanced depth imaging-optical coherence tomography in patients with obstructive sleep apnea syndrome. *Arq Bras Oftalmol* **79**, 247-252, doi:10.5935/0004-2749.20160070 (2016).
